# Navigating the Unknown: A Comprehensive Review of Spaceflight-Associated Neuro-Ocular Syndrome

**DOI:** 10.7759/cureus.53380

**Published:** 2024-02-01

**Authors:** Abhidnya Mehare, Swarupa Chakole, Bhushan Wandile

**Affiliations:** 1 Obstetrics and Gynecology, Jawaharlal Nehru Medical College, Datta Meghe Institute of Higher Education and Research, Wardha, IND; 2 Community Medicine, Jawaharlal Nehru Medical College, Datta Meghe Institute of Higher Education and Research, Wardha, IND; 3 Medicine, Jawaharlal Nehru Medical College, Datta Meghe Institute of Higher Education and Research, Wardha, IND

**Keywords:** artificial gravity, preventive measures, ocular health, astronaut health, microgravity, spaceflight-associated neuro-ocular syndrome

## Abstract

Spaceflight-associated neuro-ocular syndrome (SANS) is a complex and multifaceted condition that affects astronauts during and after their missions in space. This comprehensive review delves into the various aspects of SANS, providing a thorough understanding of its definition, historical context, clinical presentation, epidemiology, diagnostic techniques, preventive measures, and management strategies. Various ocular and neurological symptoms, including visual impairment, optic disc edema, choroidal folds, retinal changes, and increased intracranial pressure, characterize SANS. While microgravity is a primary driver of SANS, other factors like radiation exposure, genetic predisposition, and environmental conditions within spacecraft contribute to its development. The duration of space missions is a significant factor, with longer missions associated with a higher incidence of SANS. This review explores the diagnostic criteria and variability in SANS presentation, shedding light on early detection and management challenges. The epidemiology section provides insights into the occurrence frequency, affected astronauts' demographics, and differences between long-term and short-term missions. Diagnostic tools, including ophthalmological assessments and imaging techniques, are crucial in monitoring astronaut health during missions. Preventive measures are vital in mitigating the impact of SANS. Current strategies, ongoing research in prevention methods, lifestyle and behavioral factors, and the potential role of artificial gravity are discussed in detail. Additionally, the review delves into interventions, potential pharmacological treatments, rehabilitation, and long-term management considerations for astronauts with SANS. The conclusion underscores the importance of continued research in SANS, addressing ongoing challenges, and highlighting unanswered questions. With the expansion of human space exploration, understanding and managing SANS is imperative to ensure the health and well-being of astronauts during long-duration missions. This review is a valuable resource for researchers, healthcare professionals, and space agencies striving to enhance our knowledge and address the complexities of SANS.

## Introduction and background

Spaceflight-associated neuro-ocular syndrome (SANS) is a complex and intriguing medical condition that has garnered increasing attention within space exploration. SANS is a constellation of neuro-ocular findings that affect astronauts during extended space missions. The syndrome primarily manifests as a set of visual and neurological impairments and has raised concerns about the potential long-term consequences for individuals venturing beyond Earth's orbit [1]. Awe-inspiring achievements and remarkable scientific discoveries mark the history of space travel. However, it is also punctuated by unforeseen challenges and health risks associated with prolonged exposure to the microgravity environment of space. SANS, though relatively recently recognized and named, has been lurking in the background of space exploration since its earliest days [2].

The discovery and understanding of SANS have evolved alongside humanity's efforts to explore the cosmos. The syndrome's roots can be traced to observations of astronauts' visual and ocular changes during and after space missions. These observations became more pronounced during long-duration missions aboard the International Space Station (ISS). Early space travelers had experienced transient visual disturbances, but the sustained presence of astronauts on the ISS brought the syndrome into sharp focus [3].

This comprehensive review aims to shed light on SANS, delving deep into its clinical presentation, pathophysiology, epidemiology, and implications for future space exploration. We will explore the syndrome's origins, clinical manifestations, and ongoing research efforts to understand, mitigate, and ultimately prevent it. By synthesizing the existing knowledge and research, this review intends to provide a holistic overview of SANS for researchers, space agencies, and anyone intrigued by the intersection of space travel and human health.

## Review

Understanding spaceflight physiology

Overview of Spaceflight Conditions and Challenges

Spaceflight presents an extraordinary set of environmental conditions and challenges markedly different from what we encounter on Earth. Astronauts embarking on missions beyond our planet must confront the harsh realities of space, including microgravity, extreme radiation exposure, isolation, and confinement. Understanding these conditions is paramount to comprehending the development of SANS [4].

Space environments entail a unique set of challenges. Astronauts are subjected to prolonged weightlessness, isolation, and confinement, which can induce a range of physiological and psychological stressors. Prolonged missions, such as those to the ISS or future deep space endeavors, can extend for months or even years, placing astronauts at risk of developing rare health issues on Earth [5].

Effects of Microgravity on the Human Body

One of the most distinctive features of spaceflight is the absence of gravity, resulting in a state known as microgravity. This condition has profound effects on the human body. Muscles atrophy, bones weaken, and fluids redistribute throughout the body. The cardiovascular, musculoskeletal, and immune systems undergo significant adaptations, and these changes are not limited to the body's structural and functional aspects. They extend to the neuro-ocular system, which plays a pivotal role in developing SANS [6]. As astronauts experience the sensation of weightlessness, it triggers a cascade of physiological responses that can impact ocular health and the nervous system. In microgravity, bodily fluids shift toward the head, leading to increased intracranial pressure and affecting the structure and function of the eyes [7].

Role of Gravity in Ocular Health

Gravity has long been an evolutionary force shaping the biology of living organisms on Earth. It is crucial in maintaining various physiological processes, including ocular health. The eyes are intricately connected to the body's gravitational perception and rely on gravity to function optimally [8]. Gravity's role in ocular health is underscored by its involvement in regulating intraocular pressure, aqueous humor dynamics, and even the alignment of the eyes. It also assists in maintaining the structural integrity of the eye. The absence of gravity or exposure to microgravity can disrupt these finely tuned mechanisms, leading to changes central to SANS' development [9].

Factors Contributing to SANS

SANS is a complex condition influenced by a combination of factors. At its core, the effects of microgravity are pivotal, as the absence of gravitational forces significantly alters the distribution of bodily fluids, potentially leading to increased intracranial pressure and subsequent ocular health effects. However, SANS development is not solely attributed to microgravity; other variables also come into play. Radiation exposure poses a concern, particularly for prolonged space missions beyond Earth's protective magnetosphere. Genetic predisposition may explain why some astronauts are more susceptible to SANS than others, even under similar spaceflight conditions [10].

Moreover, environmental conditions within the spacecraft, including lighting, air quality, and nutrition, may influence the syndrome's development. The duration of space missions is another critical factor, as more extended missions expose astronauts to the space environment and its physiological challenges for extended periods, amplifying the risk of SANS. Microgravity-related factors beyond fluid shifts, such as muscle atrophy, can indirectly contribute to the syndrome's development. Comprehending the interplay of these multifaceted factors is imperative for developing effective preventive measures and interventions to safeguard astronaut health during space missions, making SANS research and management an ongoing priority for space agencies [11].

Clinical presentation of SANS

Signs and Symptoms

Visual impairment: Astronauts in space may experience various forms of visual impairment, including blurred vision, difficulty focusing, and changes in visual acuity. These visual disturbances can significantly impact their ability to perform tasks essential for the mission's success. Blurring vision and difficulty focusing can hinder the precision required for operating equipment, reading instruments, and spacewalks. Maintaining clear vision is vital for the safety and efficiency of astronauts in the space environment [7].

Optic disc edema: Optic disc edema, characterized by swelling of the optic nerve head, is a prominent and defining sign of SANS. This condition is typically observed in astronauts during or after space missions. The optic nerve head swelling indicates elevated intracranial pressure, which can lead to visual disturbances and is one of the primary diagnostic criteria for SANS. Detecting optic disc edema is crucial for diagnosing the syndrome and initiating appropriate intervention to preserve ocular health [12].

Choroidal folds: Choroidal folds are abnormalities characterized by the folding of tissue layers in the vascular layer of the eye, known as the choroid. These folds can lead to visual disturbances and are often detected through advanced imaging studies, such as optical coherence tomography (OCT). Choroidal folds are significant because they can further impede an astronaut's visual acuity and contribute to the overall ocular changes associated with SANS [13].

Retinal changes: SANS can result in retinal vasculature alterations and development of retinal folds. These changes affect the health and function of the retina, the light-sensitive tissue at the back of the eye. Retinal abnormalities can further compromise an astronaut's visual capabilities, making detecting and monitoring these changes essential to ensure early intervention and preserve visual health [1].

Increased intracranial pressure: Elevated intracranial pressure is a crucial mechanism underlying the development of SANS. This increased pressure within the skull can lead to various symptoms, including headaches, intracranial hypertension (increased pressure within the cranium), and pulsatile tinnitus (a rhythmic ringing or whooshing sound in the ears). These symptoms are significant indicators of the syndrome and often prompt further investigation into an astronaut's ocular and neurological health. Monitoring intracranial pressure and addressing related symptoms are essential to SANS diagnosis and management [14].

Diagnostic Criteria

Ophthalmological examination: A comprehensive ophthalmological examination is a cornerstone for diagnosing SANS. This evaluation involves a detailed assessment of astronauts' ocular health, including visual acuity measurements, intraocular pressure (IOP), and a thorough optic nerve and retina examination. Visual acuity tests, such as Snellen charts or Landolt rings, assess an astronaut's ability to see and focus accurately. Monitoring IOP is crucial for identifying changes associated with increased intracranial pressure. An in-depth examination of the optic nerve and retina using ophthalmoscopes and other specialized tools helps detect signs of SANS-related changes, such as optic disc edema, choroidal folds, and retinal abnormalities. These assessments are central to diagnosing SANS and guide subsequent evaluations and interventions [15].

Imaging studies: Various imaging techniques are instrumental in diagnosing SANS. OCT provides high-resolution, cross-sectional visualization of the retina, optic nerve, and other ocular structures, enabling the detection of structural changes associated with SANS. MRI captures detailed images of the brain and eye structures, offering insights into potential intracranial and optic nerve abnormalities. Ultrasound is valuable for assessing changes in the posterior segment of the eye when other imaging methods may be challenging to implement. These imaging studies provide essential visual and structural data, diagnosing SANS and monitoring its progression [16].

Neurological assessment: A neurological assessment is crucial for diagnosing SANS, as it helps evaluate neurological symptoms, including signs of increased intracranial pressure. Identifying these neurological symptoms is essential for differentiating SANS from other neurological conditions and ensuring accurate diagnosis. Neurological assessments may include evaluations of headache severity, papilledema (swelling of the optic nerve head), and other neurological signs indicative of elevated intracranial pressure. This assessment is a critical component of the diagnostic process, helping healthcare professionals confirm the presence of SANS and initiate appropriate management and intervention strategies [17].

Variability in Presentation

SANS does not manifest uniformly among astronauts. The presentation can vary in severity, timing, and specific symptoms. Some astronauts develop SANS early in their missions; others experience delayed onset or milder symptoms. Understanding the range of presentations is essential for effective monitoring and treatment strategies [18]. Individual factors, mission duration, and genetic predispositions may influence variability in SANS presentation. This section will explore the nuances of how SANS manifests and progresses in astronauts, highlighting the challenges in predicting and managing the syndrome [19].

Associated Risk Factors

Mission duration: The duration of a space mission is a critical factor in the development of SANS. The longer an astronaut spends in space, the higher the risk of developing SANS. This is particularly concerning for future deep space missions, such as those to Mars, which involve extended durations of exposure to microgravity. Prolonged missions increase the likelihood of experiencing physiological changes, including alterations in intracranial pressure and ocular health, which are associated with SANS. Managing and mitigating the impact of long-duration missions is crucial for preserving astronaut health and safety [19].

Genetic predisposition: Genetic predisposition is essential in SANS research. Some astronauts may be more susceptible to the syndrome due to their genetic makeup, which can influence how their bodies respond to the space environment. Variations in genes related to ocular and neurological health, fluid regulation, or inflammation may contribute to an individual's risk of developing SANS. Identifying genetic factors associated with SANS susceptibility can inform personalized risk assessments for astronauts and help space agencies tailor health management strategies accordingly [20].

Radiation exposure: Prolonged space missions, especially those beyond Earth's protective magnetosphere, expose astronauts to elevated levels of cosmic radiation. This exposure may contribute to ocular and neurological changes, further complicating the development of SANS. Understanding the impact of radiation on astronaut health, mainly its role in SANS, is crucial for planning deep space missions and implementing radiation protection measures to minimize potential risks [21].

Environmental factors: The spacecraft environment, including lighting conditions, air quality, and nutrition, can influence the development of SANS. For example, inadequate lighting conditions or suboptimal nutrition may exacerbate physiological changes associated with space travel, impacting ocular and neurological health. Assessing and optimizing environmental factors on spacecraft are essential for reducing the risk of SANS and enhancing the well-being of astronauts during space missions [22].

Epidemiology and incidence

Frequency of Occurrence in Different Space Missions

Mission duration: The duration of a space mission plays a significant role in the occurrence of SANS. More extended missions, such as those aboard the ISS or future deep space missions, often witness a higher incidence of SANS than shorter orbital missions. Extended exposure to microgravity and other space-related factors increases the likelihood of developing SANS. Prolonged missions result in more substantial changes in bodily fluid distribution and ocular parameters, increasing the risk of SANS-related symptoms. Understanding the relationship between mission duration and SANS is essential for planning and implementing preventive measures for astronauts on extended missions [23].

Mission specifics: Variations in the design and objectives of space missions can also affect the occurrence of SANS. Factors like mission profiles, onboard activities, and living conditions can contribute to differences in the incidence of SANS. For instance, missions with different physical activity levels or radiation exposure may impact the syndrome's development and severity. Examining the specific mission parameters and how they influence SANS is crucial for tailoring preventive strategies and improving astronaut health [18].

Preventive measures: Implementing specific countermeasures and preventive strategies may significantly impact the occurrence of SANS. The assessment of the effectiveness of these interventions is crucial for future space missions. This includes in-flight exercise regimens, nutritional interventions, visual therapy, and artificial gravity. Evaluating the outcomes of these preventive measures allows space agencies to refine and optimize their approaches to reduce the risk of SANS during missions. By continuously assessing the impact of preventive measures, space agencies can enhance astronaut well-being and safety during space travel [24].

Demographics of Affected Astronauts

Age: Age is a factor that may play a role in the development of SANS. Research suggests that older astronauts may be at a higher risk due to age-related changes in ocular and neurological health. The physiological changes associated with aging, such as alterations in fluid balance, vascular function, and tissue resilience, can interact with microgravity's effects and potentially increase older astronauts' susceptibility to SANS. Understanding the influence of age is vital for tailoring preventive and management strategies, particularly for missions involving older crew members, and for implementing age-specific health monitoring protocols [25].

Sex: Sex-related differences in the occurrence and severity of SANS may exist, and understanding these differences is essential for tailoring preventive and management strategies. These differences may be linked to variations in hormonal profiles, anatomical features, or genetic factors that can influence an astronaut's susceptibility to SANS. By investigating potential sex-related disparities, space agencies can adapt health monitoring and intervention strategies to account for male and female astronauts' unique needs and ensure equitable ocular and neurological health protection [26].

Health status: The health status of astronauts, including pre-existing medical conditions and a history of ocular issues, may impact their susceptibility to SANS. Individuals with specific medical conditions or prior ocular health problems may be at a heightened risk of developing SANS-related symptoms or experiencing more severe effects. Understanding the health status of affected individuals is necessary for accurate epidemiological assessments. By comprehensively evaluating the medical history and health status of astronauts, space agencies can better identify at-risk individuals and tailor preventive measures and management strategies to their specific needs, ensuring the overall well-being of astronauts during space missions [27].

Long-Term Versus Short-Term Missions

Long-term missions: SANS is more frequently observed in astronauts participating in extended missions, such as those on the ISS, which can last several months or over a year. Understanding the prevalence and progression of SANS in long-term missions is paramount for planning future deep space missions, including those to Mars and beyond. The extended duration of these missions exposes astronauts to prolonged microgravity, making them more susceptible to the physiological changes associated with SANS. Investigating the syndrome in the context of long-term missions helps space agencies develop comprehensive strategies for SANS prevention, management, and the preservation of astronaut health during extended space travel [1].

Short-term missions: Short-duration missions, such as those to the Moon or low Earth orbit, typically exhibit a lower incidence of SANS. Exploring the reasons for this difference is essential for developing preventive measures that apply to a broader range of spaceflight scenarios. These shorter missions expose astronauts to microgravity for a relatively limited period, which may reduce the risk of developing SANS-related symptoms. Analyzing the factors contributing to the lower incidence of SANS in short-term missions can provide valuable insights into the mechanisms of the syndrome and guide the development of preventive strategies that can be applied to various spaceflight scenarios. Additionally, this research can help identify specific risk factors and protective measures that are particularly relevant for shorter missions [28].

Clinical evaluation and monitoring

Ophthalmological Assessment

Visual acuity testing: Regular measurements of astronauts' visual acuity are fundamental for tracking changes in their ability to see and focus accurately. Astronauts undergo visual acuity testing using standardized methods like Snellen charts, Landolt rings, or other visual acuity charts. These tests evaluate an astronaut's ability to discern and identify symbols or letters at various distances. Frequent visual acuity assessments help healthcare professionals detect any decline in vision and identify early signs of SANS, allowing for timely intervention and management [29].

Intraocular pressure measurement: Monitoring intraocular pressure is crucial for identifying the development of conditions like glaucoma or other ocular issues related to elevated pressure within the eye. Astronauts undergo intraocular pressure measurement using techniques like tonometry. Regular assessments help healthcare professionals recognize any changes in intraocular pressure that may indicate SANS-related effects, particularly optic disc edema or other pressure-related symptoms [30].

Optic nerve examination: A detailed optic nerve examination is performed to detect signs of optic disc edema, choroidal folds, or other structural changes associated with SANS. Healthcare professionals use ophthalmoscopes, fundus photography, or OCT to visualize the optic nerve and evaluate its health. This examination is vital for identifying SANS-related ocular changes, and its findings guide further assessments and intervention strategies [31].

Retinal valuation: Astronauts undergo retinal imaging and examination to detect abnormalities such as retinal folds and changes in the retinal vasculature. Techniques like fundus photography, fluorescein angiography, and retinal OCT capture detailed retina images and identify SANS-related retinal changes. These evaluations are crucial for monitoring the retinal health of astronauts, detecting any signs of the syndrome, and helping healthcare professionals make informed decisions regarding intervention and treatment [27].

Imaging Techniques for Ocular Health Evaluation

Optical coherence tomography: OCT is a high-resolution imaging method that has proven to be indispensable in the assessment of SANS. It provides remarkable detail for non-invasive, cross-sectional visualization of the retina, optic nerve, and other ocular structures. OCT provides precise retinal and optic nerve parameter measurements, making it instrumental in detecting SANS-related changes. This imaging technique can identify subtle alterations in the thickness of retinal layers, optic nerve head morphology, and the presence of fluid accumulations, all of which are vital indicators of SANS-related ocular changes [16].

Magnetic resonance imaging: MRI is a valuable diagnostic tool for evaluating SANS. It captures detailed images of the brain and the structures surrounding the eyes, offering insights into the structural changes associated with the syndrome. MRI can reveal alterations in the optic nerve, intracranial structures, and cerebrospinal fluid dynamics. It is beneficial in assessing brain and optic nerve changes that may result from elevated intracranial pressure, providing valuable information for SANS diagnosis and management [32].

Ultrasound: Ocular ultrasound is a versatile and valuable tool for assessing changes in the posterior segment of the eye. In cases where other imaging methods, such as OCT or MRI, may be challenging to implement or unavailable, ultrasound offers a portable and practical alternative. Ocular ultrasound can provide essential information about ocular structures, vitreous changes, and potential abnormalities, particularly in astronauts with SANS-related ocular symptoms. This method is beneficial when immediate evaluation and monitoring are needed [33].

Other Diagnostic Tools

Cerebrospinal fluid analysis: Occasionally, cerebrospinal fluid (CSF) analysis may be necessary to detect elevated intracranial pressure and provide insights into SANS progression. CSF analysis can reveal the presence of abnormalities, such as increased pressure or changes in CSF composition, which may be associated with SANS-related symptoms, particularly those involving elevated intracranial pressure and the potential for optic disc edema. This diagnostic tool plays a crucial role in confirming SANS and understanding its physiological underpinnings, aiding in developing tailored intervention strategies for affected astronauts [34].

Visual field testing: Visual field testing is a valuable diagnostic method for assessing the extent and location of visual deficits. This test helps identify peripheral vision deficits, which can indicate SANS-related changes affecting an astronaut's visual field. It is beneficial in detecting conditions like optic nerve damage and choroidal folds, which may not be readily apparent through other clinical assessments. By mapping out visual field abnormalities, healthcare professionals better understand the impact of SANS on an astronaut's vision. They can tailor intervention strategies to address specific visual deficits [35].

Electroretinography (ERG): ERG is a specialized diagnostic tool that measures the retina's electrical activity. This test provides valuable insights into the functionality of retinal cells and their response to light stimuli. ERG is particularly helpful in assessing the integrity of the retinal photoreceptors, which are crucial for normal vision. It aids in identifying any disruptions in retinal function that may be associated with SANS. Changes in ERG readings can signal retinal abnormalities, helping clinicians detect and monitor the ocular effects of SANS. This information is essential for determining the syndrome's progression and guiding the development of targeted interventions to preserve visual health [36].

Regular Monitoring During Space Missions

In-flight evaluations: Astronauts aboard the ISS and other long-duration space missions undergo regular in-flight evaluations to assess changes in vision, optic nerve health, and other SANS-related parameters. The onboard medical team conducts these evaluations and may include a range of diagnostic tests, such as ocular examinations, visual acuity assessments, and imaging studies. In-flight monitoring aims to detect signs of SANS and related ocular or neurological changes, enabling timely intervention and treatment when necessary. In-flight evaluations are a critical component of astronaut health management, helping to ensure that ocular issues are addressed as they arise, preventing them from progressing to a more severe stage [1].

Data transmission to Earth: Real-time or near-real-time transmission of medical data from the spacecraft to Earth is essential for astronaut health monitoring during space missions. This data transmission enables healthcare professionals on Earth to monitor an astronaut's health status remotely. By receiving real-time medical data, experts can assess an astronaut's well-being, track the progression of SANS-related parameters, and guide the onboard medical team when necessary. Timely data transmission to Earth is vital for early detection and intervention, as it allows for swift medical responses and the adjustment of treatment plans, ultimately contributing to astronaut health and safety [37].

Post-mission evaluations: Upon returning to Earth, astronauts undergo comprehensive post-mission evaluations to assess the impact of spaceflight on their ocular and overall health. A team of medical professionals conducts these evaluations and may include extensive ocular examinations, imaging studies, and neurological assessments. Post-mission evaluations provide insights into the long-term effects of space travel, including any changes that may have occurred because of SANS. These assessments inform astronauts' recovery plans and help healthcare professionals tailor interventions, such as rehabilitation and visual therapy, as needed. Additionally, the data gathered during post-mission evaluations contribute to the ongoing study of SANS and its implications for future space missions [27].

Mitigation and prevention

Current Strategies to Prevent SANS

In-flight exercise: In-flight exercise is a cornerstone of preventing SANS strategies. Astronauts are prescribed exercise regimens to counteract the physiological changes brought about by prolonged exposure to microgravity. These exercises are meticulously designed to maintain astronauts' cardiovascular health, muscle mass, and bone density, indirectly contributing to ocular health. Regular physical activity helps astronauts counteract muscle atrophy, bone loss, and the fluid shift toward the head, which can lead to elevated intracranial pressure. In-flight exercise not only supports their overall physical well-being but also aids in preventing SANS by mitigating several of the risk factors associated with the syndrome [1].

Nutritional interventions: Dietary adjustments and nutritional supplements are vital in supporting astronauts' ocular and overall health during extended space missions. Specialized diets may include antioxidants, vitamins, and nutrients that have the potential to protect ocular tissues from the adverse effects of microgravity. These nutritional interventions are designed to address the unique challenges of space travel and minimize the risk of ocular changes associated with SANS. Researchers and nutritionists work to optimize astronaut diets to ensure they receive the necessary nutrients while investigating the potential benefits of specific dietary components in preventing or mitigating the syndrome [22].

Hydration management: Proper hydration is essential for managing intracranial pressure and preventing the development of optic disc edema, a key feature of SANS. Astronauts are encouraged to maintain adequate fluid intake throughout their missions to ensure their bodies remain hydrated. This helps regulate fluid balance and prevents excessive fluid shift toward the head, which can contribute to increased intracranial pressure. Monitoring hydration status is essential to mission health management, and astronauts receive guidance on maintaining fluid balance in the microgravity environment. Hydration management is fundamental to preventing the ocular and neurological changes associated with SANS [38].

Research and Development in Prevention Methods

Pharmacological interventions: Research into pharmacological interventions is pivotal to SANS prevention and management. This study area is focused on investigating the potential of drugs and medications to prevent or alleviate the effects of SANS. Specifically, researchers are exploring pharmaceutical agents that target fluid shifts and intracranial pressure regulation. These interventions aim to counteract the physiological changes experienced by astronauts in microgravity, which can contribute to ocular symptoms associated with SANS. The research includes identifying and testing medications that can help maintain fluid balance, reduce intracranial pressure, and mitigate the risks of optic disc edema. Continuous efforts in pharmacological research are essential to developing effective drugs and medications to safeguard astronaut ocular and neurological health during long-duration space missions [39].

Advanced exercise technologies: Developing advanced exercise technologies is crucial for enhancing the effectiveness of in-flight exercise regimens. Exercise is a cornerstone of SANS prevention, as it helps counteract the physiological consequences of prolonged exposure to microgravity. Researchers are working on cutting-edge exercise equipment and techniques to make in-flight exercise more efficient and tailored to astronauts' needs. These technologies aim to maximize the benefits of exercise in space, promoting cardiovascular health, muscle mass, and bone density, all of which indirectly contribute to ocular health. By improving exercise regimens through advanced technologies, space agencies enhance astronauts' physical well-being, reducing the risks associated with SANS and other space-related health issues [40].

Countermeasure validation: The validation of countermeasures is an ongoing process in the field of SANS research. This entails continuous evaluation of existing countermeasures' effectiveness and adaptability to different spaceflight scenarios. Researchers and space agencies work to assess the performance of preventive strategies, such as exercise, nutritional interventions, and other measures to mitigate the impact of SANS. Through rigorous testing and validation, the scientific community ensures that these countermeasures are effective, safe, and suitable for various space missions. Given the diversity of space exploration endeavors, the adaptability of these measures to different mission types, durations, and destinations is a crucial consideration. By continuously validating countermeasures, space agencies can refine their approaches, improve astronaut health, and further the prevention and management of SANS [24].

Lifestyle and Behavioral Factors

Sleep patterns: Proper sleep patterns safeguard ocular and neurological health during extended space missions. Adequate and high-quality sleep is pivotal in regulating intracranial pressure, a critical factor in SANS. Healthy sleep patterns are encouraged to minimize the risk of elevated intracranial pressure, which can contribute to ocular symptoms such as optic disc edema. Astronauts are advised to adhere to structured sleep schedules, ensuring sufficient rest in the microgravity environment. Maintaining regular sleep patterns mitigates SANS-related risks and promotes overall well-being and cognitive function, which is crucial for mission success [41].

Psychological support: The psychological well-being of astronauts is integral to their health and performance during space missions. Psychological stressors, such as isolation, confinement, and the demands of space travel, can exacerbate the physiological changes associated with extended missions and SANS. As a result, the provision of psychological support systems is essential. Behavioral interventions, counseling, and emotional support are incorporated to assist astronauts in coping with the unique psychological challenges of long-duration space travel. These support systems aim to enhance astronauts' resilience, emotional stability, and ability to manage stress effectively. By addressing the psychological aspects of space travel, space agencies prioritize the mental health of astronauts, ensuring that they are better equipped to face the rigors of extended missions and reduce the overall risk of SANS [42].

Role of Artificial Gravity

Artificial gravity represents an innovative approach to address the adverse effects of microgravity, including SANS. This section explores the potential of artificial gravity and its ongoing research for preventing SANS [1]. Artificial gravity is a proposed solution to mitigate the physiological changes experienced by astronauts in the microgravity environment of space. It involves the creation of centrifuge-based systems or habitats that replicate the conditions of Earth's gravitational force. By doing so, it aims to counteract the deleterious effects of prolonged exposure to microgravity on human health, including SANS [43]. Artificial gravity systems operate by spinning a spacecraft or a specific section of it, creating a centrifugal force that simulates the pull of gravity. Astronauts inside such systems would experience a force acting outward from the center of rotation, effectively pressing them against the walls or floor of the spacecraft. This outward force mimics the sensation of gravity and addresses some of the critical physiological challenges associated with extended space missions [44].

One of the primary advantages of artificial gravity is its potential to maintain average fluid balance within the body, effectively mitigating the fluid shift toward the head that contributes to SANS. This shift can lead to elevated intracranial pressure and the ocular changes associated with the syndrome. The artificial gravity-induced force can help distribute bodily fluids more evenly, reducing the impact on the visual and neurological systems [45]. Research into the feasibility and effectiveness of artificial gravity as a preventive measure for SANS is an ongoing endeavor. Implementing artificial gravity in space missions, especially those involving long-duration voyages to destinations like Mars, presents various technical challenges. These challenges include the engineering and design of rotating spacecraft, the potential impact on crew health and comfort, and the logistical requirements for such systems [43].

Treatment and management

Interventions for Astronauts with SANS

Optical corrections: Astronauts who experience changes in visual acuity due to SANS may require optical corrections to enhance their vision during the mission. These corrections can take the form of prescription glasses or contact lenses. They are essential for astronauts to maintain clear and accurate vision, particularly for tasks that demand precision, such as reading instrument displays, conducting experiments, or performing maintenance on spacecraft systems. Optical corrections aim to address specific refractive errors and support astronauts in their daily work, ensuring they can carry out their responsibilities effectively and safely [25].

Pharmacological management: Pharmacological interventions may be considered case-by-case to address specific aspects of SANS, such as reducing elevated intracranial pressure. Medications for SANS management may include diuretics to regulate fluid balance, anti-inflammatory drugs to mitigate inflammation, and other targeted pharmaceuticals. The choice of medication and its dosage should be carefully tailored to the astronaut's needs and condition. Close medical monitoring is imperative to track the effectiveness of pharmacological management and assess any potential side effects [46].

Reduced workload: In response to the visual and neurological challenges presented by SANS, astronauts may be assigned tasks with reduced visual demands and physical exertion during their missions. This adaptation aims to prevent symptoms from worsening and ensure that astronauts can continue contributing to mission objectives without compromising their health. By lightening their workload and allocating less visually demanding tasks, space agencies can help astronauts manage their SANS-related symptoms and avoid unnecessary strain during the mission [15].

Ongoing monitoring: Frequent monitoring of astronauts during their mission and thorough follow-up evaluations upon their return to Earth are critical components of SANS management. In-flight evaluations track changes in ocular and neurological health in real-time, allowing for prompt adjustments to interventions and strategies. Follow-up evaluations post-mission provide valuable insights into the progression of SANS and its impact on astronauts' health. This ongoing monitoring ensures that medical professionals can adapt their approach as needed, ultimately contributing to the well-being of astronauts affected by SANS [18].

Potential Pharmacological Treatments

Diuretics: Diuretics are medications that promote diuresis or the removal of excess fluids from the body, potentially helping to reduce elevated intracranial pressure associated with SANS. While diuretics have shown promise in managing intracranial pressure, their use in the context of space missions requires careful consideration and monitoring. The potential side effects, such as dehydration and electrolyte imbalances, are of particular concern in the unique space environment, where maintaining fluid balance is crucial for astronauts' health. Therefore, diuretic therapy should be administered under close medical supervision, focusing on minimizing adverse effects and optimizing the benefits of SANS management [47].

Anti-inflammatory drugs: Medications with anti-inflammatory properties may hold the potential for managing the inflammatory processes associated with SANS. Inflammation can play a role in the development and progression of ocular and neurological changes in affected astronauts. Exploring anti-inflammatory drugs, such as corticosteroids or non-steroidal anti-inflammatory agents, presents a promising avenue for addressing this aspect of SANS. However, the choice of specific anti-inflammatory drugs, their dosages, and the duration of treatment must be carefully determined through ongoing research and clinical trials to ensure both safety and efficacy [48].

Neuroprotective agents: Research is underway to identify and develop neuroprotective drugs with the potential to mitigate optic nerve damage and prevent further deterioration in astronauts with SANS. These agents are designed to support the health and integrity of neurological tissues, particularly the optic nerve. Neuroprotection is a critical aspect of SANS management, aiming to halt or slow the progression of the syndrome's effects on an astronaut's visual and neurological function. The search for effective neuroprotective agents to preserve astronauts' visual health during and after space missions is a dynamic area of investigation [49].

Nutraceuticals: Nutraceuticals, which include specialized nutritional supplements, are being explored for their potential role in preventing or ameliorating the ocular changes associated with SANS. These supplements may contain vitamins, minerals, antioxidants, or other bioactive compounds that support eye health and overall well-being. Investigating nutraceuticals as a preventive or complementary strategy for SANS management reflects a holistic approach to astronaut health. Nutraceuticals aim to address nutritional deficiencies and enhance ocular resilience, contributing to preventing and managing SANS-related ocular changes [50].

Rehabilitation and Visual Recovery

Visual therapy: Visual therapy exercises are integral to the rehabilitation process for astronauts dealing with SANS. These exercises are designed to improve various visual functions, including visual acuity, contrast sensitivity, and eye coordination. Astronauts may engage in activities to enhance their ability to focus, track moving objects, and adjust to changes in their visual perception. Visual therapy helps alleviate symptoms related to SANS and promotes the recovery of normal visual function, ultimately enabling astronauts to perform their duties more effectively during their missions [51].

Low vision aids: The use of low vision aids is a practical approach to support astronauts in maintaining functionality despite visual impairments. These aids include magnifiers, telescopic devices, electronic visual aids, and specialized software. Low vision aids can assist astronauts in tasks such as reading instrument panels, viewing displays, and conducting experiments, which are essential in space. By providing access to these aids, space agencies ensure that astronauts can navigate their workspaces and continue their scientific and operational responsibilities [51].

Behavioral counseling: Coping with the psychological impact of SANS is an integral aspect of an astronaut's rehabilitation process. Behavioral counseling and emotional support are indispensable to ensuring astronauts' mental well-being. Astronauts may experience frustration, anxiety, or disappointment due to the challenges presented by visual changes and other SANS-related symptoms. Behavioral counseling provides a safe and supportive space for astronauts to express their emotions, learn coping strategies, and receive guidance on adapting to new circumstances. It helps them build resilience and maintain their psychological health while addressing the psychological aspects of SANS [52].

Long-Term Management Considerations

Post-mission monitoring: Regular and extended monitoring of astronauts following their return to Earth is critical in assessing the long-term consequences of SANS. Ocular and neurological health can continue to evolve after a space mission, making extended monitoring vital for early detection and management of any lingering issues. This monitoring may include periodic check-ups, ocular examinations, imaging studies, and evaluations of intracranial pressure. By tracking changes over time, medical professionals can tailor interventions to each astronaut's needs and contribute to the ongoing refinement of SANS management strategies [53].

Continued rehabilitation: Astronauts who have experienced SANS may require long-term rehabilitation and visual therapy to adapt to persistent visual changes and maintain their overall quality of life. Visual therapy exercises and interventions can help astronauts regain visual function and cope with visual impairments. These rehabilitation programs aim to enhance astronauts' daily functioning, improve their independence, and support their well-being, particularly if SANS-related ocular changes persist. Continued rehabilitation ensures that affected astronauts can lead fulfilling lives in space and upon returning to Earth [54].

Research and follow-up studies: Astronauts encountering SANS represent a unique population with valuable insights into the syndrome's long-term effects. Their participation in research and follow-up studies contributes to a deeper understanding of SANS and its impact on ocular and neurological health. By engaging in ongoing research, astronauts provide real-world data and experiences that can inform future space missions and improve the prevention and management of SANS. These studies enable scientists and healthcare professionals to refine their strategies, better protect astronauts, and further our knowledge of this space-related health concern [55].

Psychological support: SANS not only affects astronauts physically but can also have psychological implications. The psychological support component is essential for addressing SANS's emotional and psychological impact on affected individuals. Astronauts may grapple with frustration, anxiety, or disappointment due to ocular changes and other SANS-related challenges. Long-term management should encompass ongoing psychological support, including counseling and mental health services, to help astronauts cope with these emotional aspects. This support is essential for astronauts to maintain their mental well-being, resilience, and overall psychological health during and after their missions [56].

## Conclusions

In conclusion, this comprehensive review has uncovered the intricacies of SANS, revealing its close ties to the unique challenges of space exploration, particularly the effects of microgravity on the human body. The clinical presentation of SANS varies among astronauts, encompassing various ocular and neurological symptoms. Risk factors such as mission duration, genetic predisposition, radiation exposure, and spacecraft environmental conditions contribute to its complexity. Early diagnosis and continuous monitoring are pivotal for effective intervention, while current and potential preventive strategies include in-flight exercise, nutritional interventions, and ongoing research into pharmacological treatments. Additionally, artificial gravity's potential role in counteracting microgravity's physiological impacts has emerged as a significant area of interest. However, despite significant progress, critical questions and challenges still need to be answered, including understanding the underlying mechanisms of SANS, evaluating the long-term effects on astronauts, validating pharmacological interventions, and exploring the feasibility of artificial gravity in deep space missions. Given the importance of SANS for astronaut well-being and the broader implications for human health and space exploration, the need for continued research and collaboration is paramount, ensuring that our journey into space continues with confidence and an ever-deepening understanding of its complexities.
